# Functional Network of the Long Non-coding RNA Growth Arrest-Specific Transcript 5 and Its Interacting Proteins in Senescence

**DOI:** 10.3389/fgene.2021.615340

**Published:** 2021-03-10

**Authors:** Siqi Wang, Shengwei Ke, Yueming Wu, Duo Zhang, Baowei Liu, Yao-hui He, Wen Liu, Huawei Mu, Xiaoyuan Song

**Affiliations:** ^1^Hefei National Laboratory for Physical Sciences at the Microscale, CAS Key Laboratory of Brain Function and Disease, Division of Life Sciences and Medicine, School of Life Sciences, University of Science and Technology of China, Hefei, China; ^2^CAS Key Laboratory of Mechanical Behavior and Design of Materials, Department of Modern Mechanics, University of Science and Technology of China, Hefei, China; ^3^School of Pharmaceutical Sciences, Fujian Provincial Key Laboratory of Innovative Drug Target Research, Xiamen University, Xiamen, China

**Keywords:** long non-coding RNA, Gas5, RNA pull down, mouse brain tissue, brain-derived cell line, RNA-seq

## Abstract

Increasing studies show that long non-coding RNAs (lncRNAs) play essential roles in various fundamental biological processes. Long non-coding RNA growth arrest-specific transcript 5 (GAS5) showed differential expressions between young and old mouse brains in our previous RNA-Seq data, suggesting its potential role in senescence and brain aging. Examination using quantitative reverse transcription-polymerase chain reaction revealed that GAS5 had a significantly higher expression level in the old mouse brain hippocampus region than the young one. Cellular fractionation using hippocampus-derived HT22 cell line confirmed its nucleoplasm and cytoplasm subcellular localization. Overexpression or knockdown of GAS5 in HT22 cell line revealed that GAS5 inhibits cell cycle progression and promotes cell apoptosis. RNA-Seq analysis of GAS5-knockdown HT22 cells identified differentially expressed genes related to cell proliferation (e.g., DNA replication and nucleosome assembly biological processes). RNA pull-down assay using mouse brain hippocampus tissues showed that potential GAS5 interacting proteins could be enriched into several Kyoto Encyclopedia of Genes and Genomes (KEGG) pathways, and some of them are involved in senescence-associated diseases such as Parkinson’s and Alzheimer’s diseases. These results contribute to understand better the underlying functional network of GAS5 and its interacting proteins in senescence at brain tissue and brain-derived cell line levels. Our study may also provide a reference for developing diagnostic and clinic biomarkers of GAS5 in senescence and brain aging.

## Introduction

Cellular senescence refers to the cessation of normal cell division under various conditions such as cellular stress and DNA damage. Senescent cells have several characteristics, including constitutive DNA damage response, increased activity of senescence-related galactosidase, higher expression of p16 (*CDKN2A*) and p21 (*CDKN1A*), and formation of senescence-associated secretory phenotype and senescence-associated heterochromatin aggregation (Wei and Ji, 2018). Cell cycle arrest is also a key feature of cellular senescence (Calcinotto et al., 2019). Studies have shown that nuclear fiber layer forms can be used as an identifier of phenotypic senescent cells (Raz et al., 2008), at the same time, mitochondrial DNA damage causes mitochondrial dysfunction and upregulation of reactive oxygen species, which may be induced by telomere dysfunction caused cellular senescence (Passos et al., 2007). Higher oxidative status caused by mitochondrial dysfunction was reported to contribute to senescence acceleration and the age-dependent alterations in cell structure and function (Hosokawa, 2002, 2004).

The hippocampus, a brain region critically involved in learning and memory, is particularly susceptible to dysfunction during senescence (Kumar and Foster, 2019). It has been reported that senescent glutamatergic synapses contributed to the age-related cognitive impairment based on the mechanisms for age-associated changes in Ca^2+^-dependent synaptic plasticity (Kumar and Foster, 2019). Astrocytes, which are star-shaped glial cells in the brain, play critical roles in maintaining normal brain physiology during development and in adulthood (Wu et al., 2005). Studies showed that the expression level of glial fibrillary acidic protein (GFAP), a protein marker of astrocytes in the brain, was upregulated significantly in old senescence-accelerated-prone 8 mice, indicating an important role of GFAP in the age-related deficits in learning and memory (Wu et al., 2005). In addition, upregulation of senescence-associated proteins p16 and p21 was found in granule cells of the dentate gyrus in the irradiation-induced mouse hippocampus, showing a similar senescence phenotype (Cheng et al., 2017).

Long non-coding RNAs (lncRNAs), or RNAs > 200 nt, which remain untranslated although sometimes generate short peptides (Payre and Desplan, 2016), have a variety of functions, such as acting as a scaffold, bait, or signaling molecule, and playing a role through targeting in the genome by *cis* or *trans* regulation and antisense interference (Quinn and Chang, 2016). LncRNAs are widely distributed in cells, both in the nucleus and cytoplasm and even in mitochondria. In recent years, functional nuclear lncRNA has been widely reported. Xist, as a classical lncRNA in the nucleus, can cause random silencing of an X chromosome in female mammals (Plath et al., 2002). Metastasis-associated lung adenocarcinoma transcript 1 is widely expressed in normal mammalian tissues and abnormally expressed in many human malignant tumors, which plays an important role in varying degrees of tumor proliferation, apoptosis, invasion and metastasis, and drug resistance (Gutschner et al., 2013). Telomeres are transcribed to produce a lncRNA called TERRA (telomere repeating RNA). It can promote homologous recombination between telomeres, delay cell senescence, and maintain genomic instability (Bunch et al., 2019). Recently, more and more studies have found that lncRNAs play important roles in cellular senescence (Abdelmohsen et al., 2013); for example, lncRNA GUARDIN suppresses cellular senescence through upregulation of p21 (Sun et al., 2020), whereas lncRNA SENEBLOC is shown to block the induction of cellular senescence through dual mechanisms that converge to repress the expression of p21 (Xu et al., 2020), and lncRNA OVAAL blocks cell senescence by regulating the expression of CDK inhibitor p27 (Sang et al., 2018). LncRNA-OIS1 modulates oncogenic RAS-induced senescence through regulating the activation of dipeptidyl peptidase 4, which has established a role in tumor suppression (Li et al., 2018). LncRNA ANRIL inhibits senescence in vascular smooth muscle cells, possibly through regulating miR-181a/Sirt1 and inhibiting the p53-p21 pathway (Tan et al., 2019).

Growth arrest-specific transcript 5 (GAS5), a member of the lncRNA family, including many isoforms and some of which were localized in the nucleus, plays important roles in various processes. GAS5 can be folded into a secondary RNA structure, exposing the sequence similar to the glucocorticoid-receptor binding element, and inhibits glucocorticoid-mediated physiological functions by interacting with the DNA binding domain of the glucocorticoid receptor (Kino et al., 2010). Studies in human renal epithelial cell HEK293T have demonstrated that GAS5 can affect the expression of glucocorticoid-induced protein kinases by their downstream signaling molecules cIAP2 and SGK1 through competitive binding of glucocorticoid receptors and participates in the regulation of cell proliferation and cell growth (Tani et al., 2013). GAS5 can also be used as a decoy for microRNA or shear factors, simulating the adsorption of molecular sponge, and directly binds to microRNAs to regulate their downstream target genes (Huang, 2018). GAS5 can adsorb miR-106a-5p to regulate the Akt/mTOR pathway, thus participating in the development of gastric cancer (Dong et al., 2019). GAS5 can also participate in the regulation of gene translation. In retinal ganglion cells, the downregulation of GAS5 can reduce the expression of adenosine triphosphate-binding cassette transporter A1, thus inhibiting the apoptosis of retinal ganglion cells (Zhou et al., 2019).

Our previous RNA-Seq data showed that GAS5 exhibits differential expression patterns in young and old mice brain tissues, which suggested that it may play a role in brain aging (Wang et al., 2019). Thus, we conducted various experiments on mice brain tissues and brain-derived cell lines to explore the role and underlying mechanism of GAS5 in senescence and brain aging. In this study, we found that GAS5 inhibits cell cycle progression and promotes cell apoptosis in the hippocampus-derived HT22 cell line. Protein expression of p21, a senescence-associated marker protein, exhibited a negative correlation with GAS5 RNA level, indicating an antiaging effect of GAS5. RNA-Seq analysis of GAS5 KD HT22 cells identified differentially expressed genes (DEGs) related to cell proliferation biological processes (for example, DNA replication, nucleosome assembly, and DNA replication-dependent nucleosome assembly), which is consistent with our cell cycle analysis results. We also performed an RNA pull-down assay which suggested that GAS5 may participate in hippocampus senescence through interacting with protein groups that are involved in mitochondrial membrane respiratory chain reaction and regulation of the synaptic structure of neurons, which is associated with neurological diseases (such as Parkinson’s disease and Alzheimer’s disease).

## Results

### Growth Arrest-Specific Transcript 5 Is Highly Expressed in the Hippocampus Region of Old Mice

**FIGURE 1:**
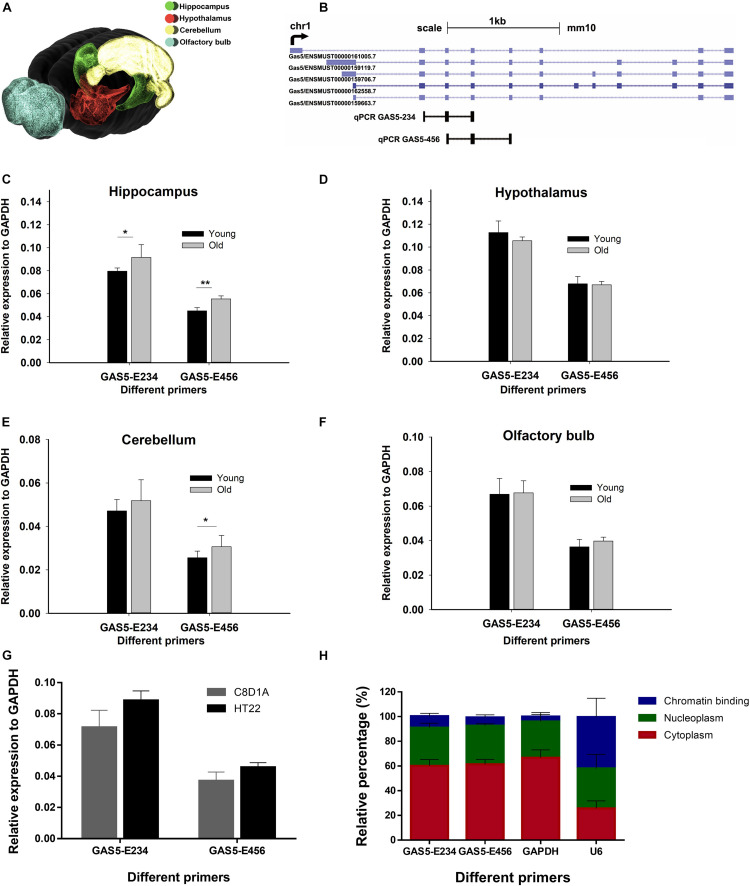
GAS5 exhibits differential expressions in different mouse brain regions of different ages. **(A)** A three-dimensional model of mouse brain showing the four regions selected for RT-qPCR. Color with green, red, yellow, and blue represent hippocampus (HC), hypothalamus (HT), cerebellum (CB), and olfactory bulb (OB), respectively. Brain model was adopted from Blue Brain Cell Atlas. Representative isoforms of GAS5 transcript were shown in **(B)** (data from University of California, Santa Cruz), and two qPCR amplicons of GAS5 (E234 and E456) were indicated below. Expression level GAS5 (represented by two pairs of primers: GAS5-E234 and GAS5-E456) in four different brain regions of young and old mice: hippocampus **(C)**, hypothalamus **(D)**, cerebellum **(E)**, and olfactory bulb **(F)**. Relative expression level of GAS5 in two brain-derived cell lines **(G)**. Subcellular localization of GAS5 in HT22 cell line **(H)**. *N* = 4 for young mice brain sample; *N* = 5 for old mice sample; *N* = 3 for **(G,H)**. *P < 0.05; **P < 0.01.

To obtain the spatial expression landscape of GAS5 in mice brain regions, we performed quantitative reverse transcription PCR (RT-qPCR) on various brain regions. Young and old male adult mice brains were dissected, and four brain regions were separated: hypothalamus (HT), olfactory bulb (OB), cerebellum (CB), and hippocampus (HC) (Figure 1A). As the GAS5 gene encodes numerous alternatively spliced lncRNA isoforms (Goustin et al., 2019), we designed two primers to cover as many GAS5 isoforms as possible for the RT-qPCR experiment: GAS5-E234 and GAS5-E456 (Figure 1B). Primer lists are shown in Supplementary Table 1. Results revealed that GAS5 showed significantly higher expression levels in old mice HC region than in young ones (Figure 1C), whereas the other three brain regions (HT, OB, and CB) did not have significant differential expression levels (Figures 1D–F). This result was consistent with the high expression level of GAS5 in a growth-arrested cell line (Plath et al., 2002). Our result also suggested that GAS5 may participate in senescence in the brain, as it showed higher expression in the aging mouse brain and also had a brain region-specific expression pattern. We also examined the expression level of GAS5 in brain-derived cell lines—C8D1A (cerebellum) and HT22 (hippocampus) (Figure 1G). Two pairs of GAS5 primers showed all high expression levels in these two cell lines, and we chose the HT22 cells to conduct the KD and OE in the following experiments because the two GAS5 primers all showed relatively consistent and high expression in HT22 cells.

To elucidate the subcellular localization of GAS5, cell fractionation was performed on HT22 cells, and we got three fractions: chromatin binding, nucleoplasm, and cytoplasm. The relative expression level (in percentage) of GAS5 and the messenger RNAs (mRNAs) of two reference genes (*GAPDH* and *U6*) in each cellular fraction was also calculated (Figure 1H). Results suggested that most of the GAS5 isoforms were mainly localized in nucleoplasm and cytoplasm.

### Growth Arrest-Specific Transcript 5 Inhibits Cell Growth and Promotes Apoptosis in Hippocampus-Derived HT22 Cells

**FIGURE 2:**
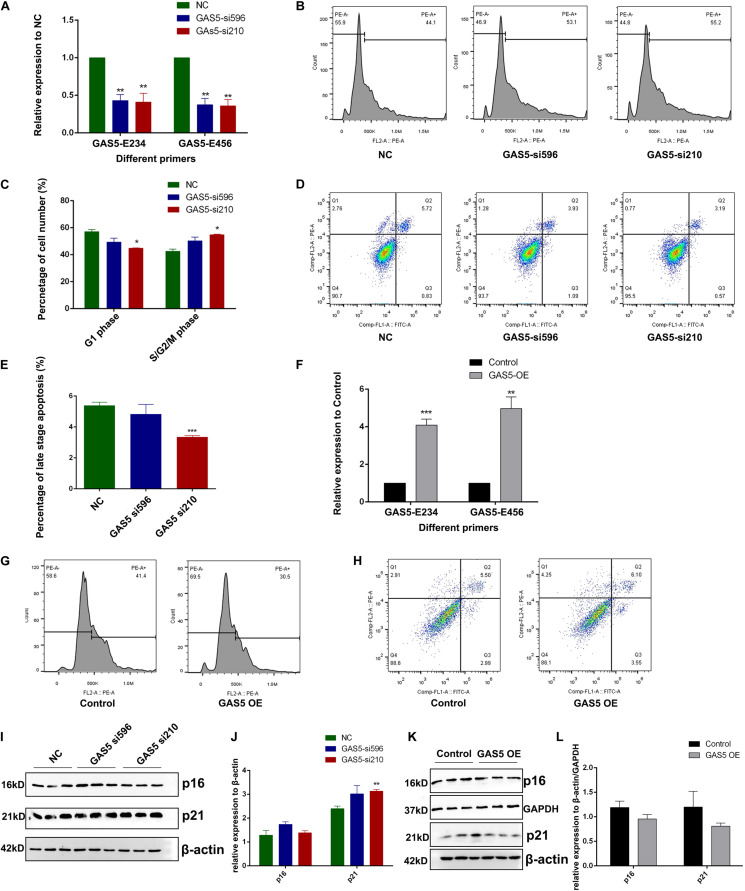
GAS5 inhibits cell growth and promotes apoptosis level in HT22 cells. **(A)** RT-qPCR was performed to quantify the relative expression of GAS5 using two different primers (GAS5-E234 and GAS5-E456) that can amplify different GAS5 isoforms in GAS5-knockdown (KD) HT22 cells. Two different siRNAs (GAS5-si596 and GAS5-si210) were used to KD GAS5. **(B)** Cell cycle analysis of GAS5 KD HT22 cell. NC, GAS5-si596, GAS5-si210 represents HT22 cells transfected with negative control siRNA, GAS5 siRNA 596, and GAS5 siRNA 210, respectively. **(C)** Percentage of cell number at G1 phase and S/G2/M phase during cell cycle was quantified. Columns in different colors represent control and GAS5 KD groups. **(D)** Cell apoptosis analysis of GAS5 KD in HT22 cells. Cells stained with negative or positive annexin V–fluorescein isothiocyanate and propidium iodide signal were separated into four parts. Top right part represents late-stage apoptosis cell. **(E)** Percentage of late-stage apoptosis cell number was quantified. Columns in different colors represent control and KD groups. **(F)** pcDNA3.1(+) vector contains GAS5 cDNA was introduced to overexpress GAS5 in HT22 cells. RT-qPCR was performed to quantify the relative expression of GAS5. **(G)** Cell cycle analysis of GAS5 overexpression (OE) in HT22 cells. **(H)** Cell apoptosis analysis of GAS5 OE HT22 cells. **(I)** Immunoblot result of p16 and p21 in GAS5-KD HT22 cells. β-actin was used as a loading control. Intensity quantification was showed in **(J)**. **(K)** Immunoblot result of p16 and p21 in GAS5 OE HT22 cells. β-actin and *GAPDH* were used as loading control. Intensity quantification was showed in **(L)**. Three biological repeats were performed in each experiment, except for **(G,H)** (*N* = 1). *P < 0.05; **P < 0.01; ***P < 0.001.

To further identify the molecular function of GAS5 in hippocampus-derived HT22 cells, we used GAS5-specific small interfering RNAs (siRNAs) (si210 and si596) to perform the KD experiment. The RT-qPCR result showed that GAS5 RNA was efficiently knocked down through siRNA compared with the control group (Figure 2A). Cell cycle analysis was performed after GAS5 KD, and results showed that depletion of GAS5 led to decreased cell number in the G1 phase (although only one siRNA group showed significance, the other siRNA group showed the same downregulation trend), whereas the cell number in S/G2/M phase was upregulated, suggesting accelerated cell cycle transition from G1 phase into the S/G2/M phase upon GAS5 KD (Figure 2B, quantification in Figure 2C). We further used annexin V–fluorescein isothiocyanate and propidium iodide (PI) to detect the apoptosis level in GAS5 KD HT22 cells. The percentage of late-stage apoptosis cells decreased after GAS5 KD (Figure 2D, quantification in Figure 2E), indicating loss of function of GAS5 inhibits cell apoptosis.

We cloned the complementary DNA (cDNA) of GAS5 into the pcDNA3.1(+) vector, and the GAS5 + pcDNA3.1(+) OE vector was introduced into HT22 cells with transfection reagent to overexpress GAS5 RNA. RT-qPCR was performed to confirm that GAS5′s RNA expression was around six times higher in the GAS5 OE group than the control group (Figure 2F). OE of GAS5 led to the opposite effect of GAS5 KD. In cell cycle analysis, cell numbers in the S/G2/M phase decreased in the GAS5 OE group, with around 10% cell arresting in the G1 phase (Figure 2G). In apoptosis assay, the portion of early- and late-stage apoptosis cells together increased upon GAS5 OE (Figure 2H).

Western blotting was performed to further detect the senescence-associated maker proteins (p16 and p21) in HT22 cells upon GAS5 KD or OE. In GAS5-KD HT22 cells, we found that p21 protein expression was upregulated (Figure 2I, quantification in Figure 2J). In the GAS5 OE group, p21 protein showed an opposite expression pattern with a lower expression level (Figure 2K, quantification in Figure 2L). However, p16 protein expression did not change significantly upon GAS5 KD but decreased in the GAS5 OE group (Figures 2I,K, quantification in Figures 2J,L). Overall, our data suggested that GAS5 inhibits cell cycle progression and promotes the apoptosis process in HT22 cells.

### RNA-Seq Revealed That Genes Affected by Downregulation of Growth Arrest-Specific Transcript 5 Might Participate in Cell Cycle

To further explore the genes that GAS5 may regulate, we performed RNA-Seq analysis using GAS5 KD HT22 cells with siRNA NC as a control. The data showed that GAS5 RNA level indeed decreased in both siRNA 210 and siRNA 596 KD HT22 cells. Using the Pearson correlation analysis method, we found that the reproducibility of the data of two siRNA KD groups was very good (*R*^2^ = 0.9965 for KD group; *R*^2^ = 0.9707 for NC group). We thus combined the data of siRNA 210 and siRNA 596 KD groups together as KD group and performed a comparison between KD and control groups.

**FIGURE 3:**
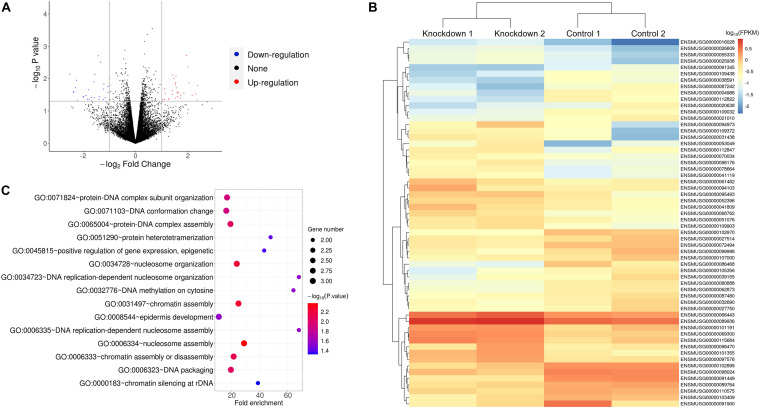
RNA-Seq analysis of HT22 cells upon GAS5 KD revealed that the affected genes might participate in cell cycle. **(A)** Volcano plot for differentially expressed genes (DEGs) between control and GAS5 KD groups. **(B)** Heatmap of the 58 DEGs with the highest significance levels between control and GAS5 KD groups. Key of heatmap represented the log_10_(FPKM) of 58 DEGs. **(C)** Gene ontology enrichment analysis for “Biological Process” of upregulated DEGs in GAS5 KD group.

The result showed that a total of 58 DEGs were identified upon GAS5 KD in HT22 cells using the criteria of *P*-value < 0.05 and fold change > 2 (Figure 3A). The heatmap of hierarchical clustering analysis indicated that all of the differentially expressed mRNAs were clustered in several groups (Figure 3B and Supplementary Table 2). We further performed gene ontology (GO) enrichment analysis to explore the biological process of the DEGs involved in. We found that 27 GO terms were significantly enriched for the upregulated DEGs (*P* < 0.05), and the top 10 enriched terms included nucleosome assembly, protein-DNA complex assembly, and DNA replication-dependent nucleosome assembly (Figure 3C and Supplementary Table 3). A previous study reported that DNA replication plays a vital role in the S phase during the cell cycle, and it is highly correlated with the protein–DNA complex during nucleosome assembly (Krude and Keller, 2001). Chromatin assembly factor-1 also plays essential roles in nucleosome assembly, which is associated with DNA replication and cell proliferation, through interacting with polymerase sliding clamp proliferating cell nuclear antigen (Takami et al., 2007). In addition to that, cell cycle machinery also showed a strong link with the nucleosome assembly activity by chromatin assembly factor-1 during DNA replication (Krude and Keller, 2001). Consistently, our data suggested that GAS5 inhibits cell cycle progression, indicating that GAS5 affects the HT22 cell cycle by regulating the mRNA level of these DEGs.

### Growth Arrest-Specific Transcript 5 May Interact With Proteins Associated With Hippocampus Function in Young Mice

**FIGURE 4:**
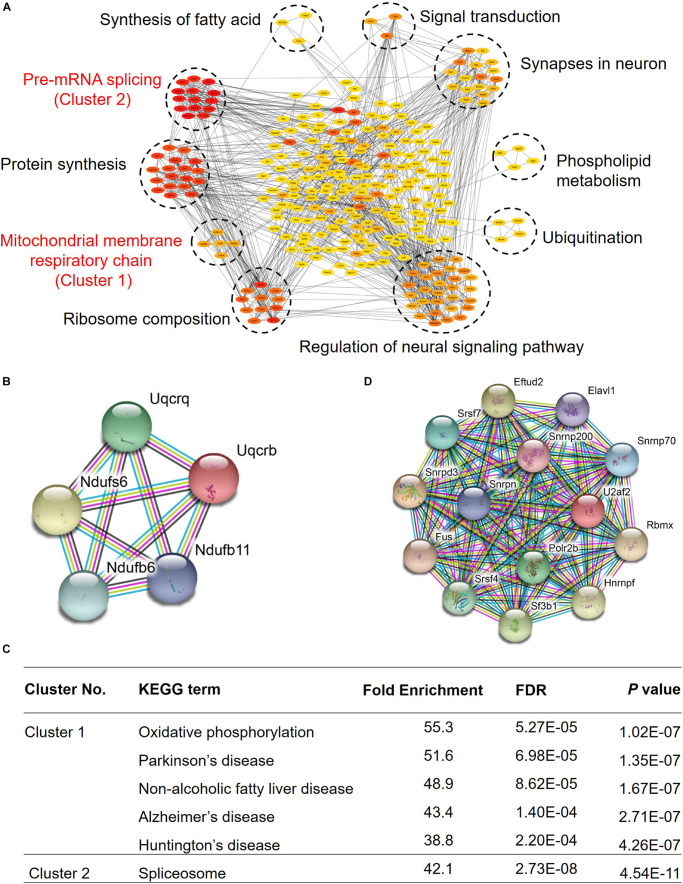
GAS5 RNA pull-down results revealed regulatory GAS5-associated protein–protein interaction network related to aging-associated diseases. **(A)** Overall protein–protein interaction network of GAS5 interacting proteins. Network nodes of different colors present proteins. Red color represents strong interaction, whereas yellow suggests a relatively weak connection. Lines between proteins represent protein–protein associations. **(B)** Interaction network diagram of proteins in first cluster, which were involved in the formation of mitochondrial membrane respiratory chain reaction. **(C)** KEGG pathway enrichment analysis of clusters 1 and 2. **(D)** Interaction network diagram of proteins in second cluster, which participated in the shearing process of pre-mRNA.

Growth arrest-specific transcript 5 was reported to play a very important role in tumors, and it can inhibit migration and invasion of gastric cancer *via* interacting with p53 protein (Liu et al., 2020), and it can also regulate the expression of p21 to enhance G1 cell cycle arrest through binding to Y-box binding protein 1 (Liu et al., 2015). To explore the role of GAS5 in the mouse hippocampus, we conducted an RNA pull-down assay in the mouse hippocampus, exploring its potential interacting proteins. Our data showed that GAS5 may interact with 290 proteins, which can form approximately 10 clusters, and they are involved in a variety of physiological processes in hippocampal brain tissue, indicating that GAS5 has multiple functions in the brain (Figure 4A and Supplementary Table 4), for example, the pre-mRNA splicing and extension of protein synthesis, mitochondrial membrane respiratory chain and ribosome composition, regulation of neural signaling pathways and ubiquitination, phospholipid metabolism and synapses in neurons, signal transduction, and synthesis of fatty acids.

Kyoto Encyclopedia of Genes and Genomes (KEGG) pathway analysis indicated that two clusters showed relatively strong interaction with GAS5, and they were taken into account for further analysis. In the first cluster, the proteins encoded by these five genes are mainly involved in the mitochondrial membrane respiratory chain (Figure 4B). KEGG pathway enrichment analysis showed that they are not only involved in oxidative phosphorylation but also participate in some important neurological diseases, such as Parkinson’s disease and Alzheimer’s disease (Figure 4C). We thus speculated that GAS5 might play a role in some neurological diseases. Proteins of the second cluster interact strongly with each other (Figure 4D). They mainly function in the shearing process of pre-mRNA (Figure 4C). Taken together, our results showed that GAS5 potentially regulates mitochondrial function, neurological diseases, and synapse function through interacting with specific protein clusters in the mouse brain hippocampus.

## Discussion

Growth arrest-specific transcript 5 was first discovered in 1988 by Schneider et al. (1988) in mouse NIH/3T3 fibroblast due to its increased expression after cell growth stagnation. As the research on GAS5 going further, more and more studies have shown that lncRNA GAS5 is low-expressed in most of the tumor samples, such as glioma, non-small cell lung cancer, breast carcinoma, etc. As a tumor suppressor, GAS5 is involved in the regulation of tumor occurrence and progression (Cao et al., 2017; Gu et al., 2018; Liu et al., 2018). However, it was not clear whether it plays a role in senescence or aging.

Our previous RNA-Seq data showed that GAS5 had differential expression patterns in young and old mice brains, which suggested that it may participate in brain aging (Wang et al., 2019). Further examination using RT-qPCR on four different brain tissues confirmed that GAS5 is expressed significantly higher in the old mouse brain hippocampus region comparing with that of a young mouse. To explore the role and underlying mechanism of GAS5 in senescence and brain aging, we performed a series of experiments on mice brain tissues and brain-derived cell lines. GAS5 showed nucleoplasm and cytoplasm subcellular localization.

Cellular senescence is an effective barrier to tumorigenesis *in vivo* (Braig et al., 2005; Collado et al., 2005; Michaloglou et al., 2005). GAS5 can inhibit bladder cancer cells proliferation and promote apoptosis (Chen et al., 2020) and also inhibits migration of gastric carcinoma cell *via* interacting with p53 protein (Liu et al., 2020). Our data showed that declined expression of GAS5 could activate cell cycle progression and reduce cell apoptosis level, and OE of GAS5 led to the opposite effect. p16 is a senescence-associated marker, the expression of which increases exponentially with aging in most tissues (He and Sharpless, 2017). p21 is a cyclin-dependent kinase inhibitor, which can also be used to measure the levels of aging (Wang et al., 2017). Upregulation of senescence-associated proteins p16 and p21 was found in granule cells of the dentate gyrus in the irradiation-induced mouse hippocampus, showing a similar senescence phenotype (Cheng et al., 2017). In our immunoblot result, the expression of p21 was upregulated upon GAS5 KD in HT22 cells and decreased in the presence of GAS5, indicating the antiaging effect of GAS5 in HT22 cells probably through the p21 pathway, although p16 protein expression did not change due to alteration of GAS5 RNA.

Growth arrest-specific transcript 5 can competitively bind Smad3 proteins through a variety of RNA SMAD-binding elements to inhibit transforming growth factor/Smad3-mediated smooth muscle cell differentiation (Tang et al., 2017), and subsequent experiments have found that GAS5 can inhibit the expression of adenosine triphosphate-binding cassette transporter A1 by binding to the enhancer of zeste homolog 2 (Meng et al., 2020). Our RNA pull-down assay of mouse hippocampus tissue identified a lot of new GAS5 interacting proteins. Mitochondrial DNA damage causes mitochondrial dysfunction and upregulation of reactive oxygen species, which may be induced by telomere dysfunction-caused cellular senescence (Passos et al., 2007). Mitochondrial dysfunction has been implicated in the pathogenesis of many neurodegenerative diseases, such as Parkinson’s disease, Huntington’s disease, and Alzheimer’s disease (Aliev et al., 2004). It is also believed that higher oxidative status caused by mitochondrial dysfunction contributes to senescence acceleration and the age-dependent alterations in cell structure and function (Hosokawa, 2002, 2004). In our study, in addition to one cluster with the strongest interactions involved in pre-mRNA splicing and extension of protein synthesis, one cluster (Uqcrq, Uqcrb, Ndufb6, Ndufb11, and Ndufs6) is involved in mitochondrial membrane respiratory chain reaction. KEGG pathway enrichment analysis showed that they are involved in some important neurological diseases, such as Parkinson’s disease, Alzheimer’s disease, Huntington’s disease, and so on. These data imply that GAS5 may have a potential role in brain aging through regulating the mitochondrial function in the hippocampus with specific mitochondrial proteins.

Growth arrest-specific transcript 5 has been reported to participate in several pathways such as apoptosis and cell cycle in T cell line and tumor suppression in many neoplasms. Our study reports the potential roles of GAS5 in senescence and brain aging. It may interfere with cell cycle, progression, and proliferation through interacting with various proteins and affecting mRNAs expression at brain tissue and brain-derived cell line levels. This study could provide a reference for further studies on molecular functions of GAS5 as well as its interacting proteins and affected mRNAs in aging and also contribute to better understand the multiple roles of non-coding RNAs in various biological systems.

## Materials and Methods

### Animal Experiments

All the animal experiments performed in this study were conducted in adherence with guidelines of University of Science and Technology of China Animal Resources Center and University Animal Care and Use Committee. Male C57BL/6 mice were housed in a specific-pathogen-free and temperature-controlled room with a 12-h light/dark cycle and unrestricted access to food and water. Mouse experiment was approved by the Committee on the Ethics of Animal Experiments of the USTC (Permit Number: USTCACUC1801051).

Young and old adult male mice were anesthetized and transcardially perfused with 1 × phosphate-buffered saline (PBS). Brain tissues from the HT, OB, CB, and HC were carefully dissected under a stereomicroscope and then quickly immersed into TRIzol Reagent (Ambion, 15596018, Life Technology, United States) on ice, which will be stored in −80°C and used for RT-qPCR experiment.

### Cell Culture

HT22 (hippocampal neuronal cell line) was routinely cultured in Dulbecco’s modified Eagle medium (Gibco, 12100046, Thermo Fisher Scientific, United States) with 10% fetal bovine serum (ExCell Bio, FSS500, China), antibiotic–antimycotic (Wisent, 450-115-EL, Nanjing, China), and incubated in a humidified atmosphere of 5% CO_2_ and 95% air at 37°C. The cells were subcultured every 2 days.

### Quantitative Reverse Transcription PCR

Total RNA from four brain regions (HT, OB, CB, and HC) were extracted using TRIzol Reagent (Ambion, 15596018, Life Technology, United States). RNA quality and quantity were checked by agarose gel electrophoresis and Nano-300 Micro Spectrophotometer (ALLSHENG, China). The first-strand cDNA was reverse transcribed using a HiScript II 1st Strand cDNA Synthesis Kit (Vazyme, R212-02, Nanjing, China) according to the manufacturer’s instructions. qPCR was then performed on a Bio-Rad CFX96 qPCR machine using the AceQ qPCR SYBR Green Master Mix (Vazyme, Q111-03, Nanjing, China). The expression levels of target genes were normalized to a housekeeping gene (*GAPDH*). Negative controls and technical replicates were also conducted. Primer lists are shown in Supplementary Table 1.

### Fractionation of Cells

Cells with a density of 10^6^ were rinsed three times with 1 × PBS and resuspended with RSB-100 solution [10-mM Tris–hydrochloric acid, pH = 7.4; 100-mM sodium chloride (NaCl); 25-mM magnesium chloride (MgCl_2_); 40 μg/ml digitonin]. Gently mix the solution on ice and centrifuge at 2,000 × *g* for 8 min, 4°C. The supernatant was the cytoplasm part. Resuspend the pellets with 500 μl of RSB-100 solution containing 0.5% Triton X-100, incubated the samples on ice for 5 min, and then centrifuged at 2,000 × *g* for 8 min, 4°C. The supernatant was the nucleoplasm part. The left pellets, which was a chromatin-binding fraction, were dissolved using 500 μl of RSB-100 solution containing 0.5% Triton X-100. Total RNAs from three fractions were extracted using TRIzol Reagent, and RT-qPCR was conducted as mentioned above.

### Growth Arrest-Specific Transcript 5 Knockdown and Overexpression

Small interfering RNA (siRNA) was used to knock down the expression of GAS5 non-coding RNA in HT22 cells and were synthesized by GenePharma (Shanghai, China). The forward and reverse sequences of GAS5-siRNA210 were 5′-CCU CUG UGA UGG GAC AUC UTT-3′ and 5′-AGA UGU CCC AUC ACA GAG GTT-3′ (Sun et al., 2017), respectively. The forward and reverse sequences of GAS5 siRNA596 were 5′-CCG GUC CUU CAU UCU GAA UTT-3′ and 5′-AUU CAG AAU GAA GGA CCG GTT-3′, respectively. The forward and reverse sequences of negative-control siRNA (NC siRNA) were 5′-UUC UCC GAA CGU GUC ACG UTT-3′ and 5′-ACG UGA CAC GUU CGG AGA ATT-3′, respectively. Cells were seeded in 12-well culture plates and transfected with siRNA when cells had reached 30 to 40% confluences. Then, cells were transfected with 10-nM GAS5 siRNA or NC siRNA using INTERFERin reagent (Polyplus, 409-10, France) at 1 of 250 final dilutions for 48 h.

cDNA of GAS5 isoform 210 was cloned into pcDNA3.1(+) (Invitrogen) OE vector. pcDNA3.1(+) empty vector was used as a negative control. Cells were seeded in 12-well culture plates and transfected with an OE vector when cells had reached 60 to 80% confluences. Then, cells were transfected with 2-μg GAS5-iso210 pcDNA3.1(+) or empty pcDNA3.1(+) using jetOPTIMUS reagent (Polyplus, 117-01, France) at 1 of 500 final dilutions for 48 h.

### Cell Apoptosis Assay by Flow Cytometry

Cells were collected from 12-well plates at 48 h after transfection, washed with PBS, suspended with 100 μf binding buffer, and then, 5-μb annexin V–fluorescein isothiocyanate and 5-μa PI (Vazyme, A211-02, Nanjing, China) were added. The liquid was incubated in binding buffer at room temperature for 10 min, and then a 400-μl binding buffer was added to the tube. Finally, cell apoptosis was examined by flow cytometry (CytoFLEX, United States). Data were analyzed using the Flowjo VX. At least three independent experiments were conducted in the analysis.

### Cell Cycle Analysis

Cells were collected from 12-well plates at 48 h after transfection, washed with 1 × PBS, fixed in 70% ethanol, and incubated at 4°C for 30 min. Fixed cells were washed twice and resuspended in PBS containing RNase A (100 μg/ml) and PI (50 μg/ml), incubated at 4°C for 30 min. At last, cell cycle analysis was performed using flow cytometry (CytoFLEX, United States), and then data were analyzed using the Modfit. At least three independent experiments were conducted in the analysis.

### Western Blot Analysis

Cells were collected from 12-well plates at 48 h after transfection, washed with 1 × PBS, lysed in radioimmunoprecipitation assay buffer [50-mM Tris–hydrochloric acid, pH = 7.4, 150-mM NaCl, 1-mM ethylenediaminetetraacetic acid, 1% Triton X-100, 1% sodium dodecyl sulfate (SDS), 1% sodium deoxycholate supplemented with 1 × protease inhibitor cocktail, and 1-mM phenylmethylsulfonyl fluoride] at 4°C for 30 min. Protein supernatants were collected through a centrifuge at 12,000 rcf for 15 min at 4°C and mixed with protein loading buffer and denatured at 100°C for 10 min. Protein samples were separated through 12% SDS-polyacrylamide gel electrophoresis (PAGE) and transferred onto polyvinylidene difluoride membranes. After blocking non-specific binding with 3% bovine serum albumin (Sangon, China) in Tris–buffered saline with 0.05% Tween 20, the blots were incubated with specific antibodies. Commercial antibody used for blot includes p16 antibody (Abcam, ab108349), beta-actin antibody (Proteintech, 60008-1-Ig), and p21 antibodies (Millipore, 05-345). The secondary antibodies used for IB (1:5,000) were horseradish peroxidase-conjugated Affinipure Goat Anti-Mouse IgG(H + L) antibody (Proteintech, China), and the signals were detected with Supersensitive ECL chemiluminescence solution (P&Q Science Technology, Shanghai, China). The intensity of bands was assessed using Image J.

### RNA Pull-Down

Yong mice (2.5 months old) were anesthetized and transcardially perfused with 1 × PBS. Hippocampus tissue from the brain was collected for homogenization on ice for 90 s and centrifuged (700 rcf) at 4°C for 5 min. After removing the supernatant, the precipitates were resuspended in the lysis buffer containing 10 mmol/L 4-(2-hydroxyethyl)-1-piperazineethanesulfonic acid, 10 mmol/L KCl, 1.5 mmol/L MgCl_2_, and 1% NP-40 and incubated on ice for 1.5 h. During lysis, *in vitro* transcribed and biotinylated GAS5 sense and antisense RNA probes were heated in RNA structure buffer containing 10 mmol/L Tris–Cl (pH = 8.0), 0.1 mol/L KCl, 1 nmol/L, and 10 mmol/L MgCl_2_ at 95°C for 2 min and was incubated on ice for 3 min. Then sense and antisense (negative control) GAS5 RNA probes were incubated with lysate from the previous discussion for 12 h at 4°C with rotation. Next, 20-μl M-280 Streptavidin magnetic beads (Invitrogen, 11205D, Thermo Fisher Scientific, United States) were added into the mixture mentioned earlier and incubated for 3 h at 4°C with rotation. Beads were washed at least three times, and RNA–protein complex was collected through a magnetic base. Beads were resuspended in elution buffer containing 7.5 mmol/L 4-(2-hydroxyethyl)-1-piperazineethanesulfonic acid, 15 mmol/L ethylenediaminetetraacetic acid, 0.15% SDS, 75 mmol/L NaCl, 0.02% sodium deoxycholate. Finally, the eluted proteins were separated by SDS-PAGE and then identified by mass spectrometry (MS). All processes were performed under RNase-free conditions.

### Mass Spectrometry

The proteins that were separated by SDS-PAGE were reduced using 20-mM dithiothreitol (Sigma) at 95°C for 5 min and alkylated in 50-mM iodoacetamide (Sigma) for 30 min (in the dark). Then, the samples were digested into peptides using sequencing grade trypsin (Promega) and recovered from the SDS-PAGE gel. Peptides were desalted using StageTips and resuspended using 0.1% formic acid with 2% acetonitrile. Mass spectrometry experiments were performed on a nanoscale ultra-high-pressure liquid chromatography system (EASY-nLC1200, Thermo Fisher Scientific) connected to an Orbitrap Fusion Lumos equipped with a nanoelectrospray source (Thermo Fisher Scientific). The peptides were separated on a reversed-phase high-performance liquid chromatography analytical column (75 μm × 25 cm) packed with 2-μm C18 beads (Thermo Fisher Scientific) using a linear gradient ranging from 9 to 28% acetonitrile for 90 min and followed by a linear increase to 45% B for 20 min at a flow rate of 300 nl/min. The Orbitrap Fusion Lumos acquired data in a data-dependent manner alternating between full-scan MS and MS2 scans. The MS spectra (350–1,800 m/z) were collected with 120,000 resolution, automatic gain control of 4 × 10^5^, and 50-ms maximal injection time. Selected ions were sequentially fragmented in a 3-s cycle by higher-energy C-trap dissociation with 30% normalized collision energy, specified isolated windows 1.6 m/z, 30,000 resolution. Automatic gain control of 5 × 10^4^ and 100-ms maximal injection time were used. Dynamic exclusion was set to 30 s. Raw data were processed using Proteome Discoverer (PD, version 2.2), and MS/MS spectra were searched against the reviewed Swiss-Prot mouse proteome database. The cutoff values are shown later: a precursor mass tolerance of 10 ppm; fragment mass tolerance of 0.02 Da; only peptides longer than six amino acids were kept; false discovery rate <1%; and at least one unique peptide was required for protein identification. The MS data have been submitted to ProteomeXchange and available *via* ProteomeXchange with identifier PXD023363.

### RNA Sequencing

The mRNA-Seq library was prepared according to the manufacturer’s protocol. Reagents used for mRNA-seq library preparation were from New England BioLabs Company. Briefly, total RNAs were extracted by TRIzol reagent. RNA quality and concentration were checked by agarose gel electrophoresis and Nanodrop. RNA integrity was validated by Agilent 2100. The mRNAs were purified by oligo-dT attached beads and fragmented into small pieces. Cleaved RNA fragments were reverse-transcribed into first-strand cDNAs. DNA polymerase I was used to synthesizing second-strand cDNAs. End repair and ligation of adapters were performed to add a single “A” base and adapters to cDNA fragments. Purified cDNA fragments were amplified through PCR. The final cDNA library was then sequenced by high throughput sequencing platform Illumina Novaseq 6000. The RNA-Seq data have been deposited in the Gene Expression Omnibus (accession number: GSE163237).

### Bioinformatics Analysis

The RNA-Seq reads were initially subject to adapter removal and filtered using Trimmomatic (Bolger et al., 2014) (version 0.39). Then, we use FastQC (version 0.11.8) to do some quality control checks. Clean reads were mapped using HISAT2 (Kim et al., 2015) (version 2.1.0) to the GRCM38 Ensembl genome. Sort and convert the SAM files to BAM files using SAMtools (Li et al., 2009) (version 1.7). The mapped reads were then counted using featureCounts (Liao et al., 2014) (version 2.0.1) with Mus_musculus.GRCm38.101.gtf annotations^1^. DEGs were determined using R package DESeq2 (Love et al., 2014) (version 1.26.0) with a *P*-value < 0.05 and an absolute value of log_2_FC > 1. Pearson correlation analysis was conducted in R (version 3.6.3). Volcano map and heatmap generation were conducted, respectively, using R packages “ggplot2” (version 3.3.2) and “pheatmap” [Kolde R, Kolde MR. Package “pheatmap”^2^ (Oct 12; 2015)] (version 1.0.12). The interaction network was analyzed in STRING (Szklarczyk et al., 2019)^3^ and visualized in Cytoscape (Otasek et al., 2019) (version 3.8.0). We used DAVID (Huang et al., 2009)^4^ to perform functional annotation clustering with the DEGs.

### Statistical Analysis

Each experiment was repeated two or three times. Student’s *t*-tests analyzed statistical differences. Data are presented as the mean ± standard errors of the means. The statistical significance was established at 0.05 (^∗^*P* < 0.05, ^∗∗^*P* < 0.01, and ^∗∗∗^*P* < 0.001) using SPSS^®^ software V.21 (Chicago, IL, United States). GraphPad Prism 7.0 was used for statistical illustrations.

## Data Availability Statement

The raw data supporting the conclusions of this article will be made available by the authors, without undue reservation.

## Ethics Statement

The animal study was reviewed and approved by the University of Science and Technology of China Animal Resources Center and University Animal Care and Use Committee.

## Author Contributions

XS and HM conceived the experiment. SW and SK performed the gene KD and OE part. SW, HM, and BL performed the RNA pull-down experiment. Y-HH and WL conducted the protein MS part. YW conducted the bioinformatic analyses. HM, SW, SK, and DZ finished RT-qPCR and cellular localization. DZ and BL took care of the animals. All authors read, wrote, and approved the article.

## Conflict of Interest

The authors declare that the research was conducted in the absence of any commercial or financial relationships that could be construed as a potential conflict of interest.

## Footnotes

1 http://asia.ensembl.org/index.html

2 https://cran.r-project.org/web/packages/pheatmap/

3 https://string-db.org/

4 https://david.ncifcrf.gov/

## Supplementary Material

Supplementary Table 1 The sequence of RT-qPCR primer for mouse GAS5.

Supplementary Table 2 The expression level, log_2_FoldChange, and adjusted *P*-values of 58 DEGs.

Supplementary Table 3 The detailed information of GO terms showed in Figure 3C.

Supplementary Table 4 The detailed information of identified proteins in RNA pull-down experiment.
